# Respiratory viral infections are underdiagnosed in patients with suspected sepsis

**DOI:** 10.1007/s10096-017-2990-z

**Published:** 2017-05-17

**Authors:** L. R. Ljungström, G. Jacobsson, B. E. B. Claesson, R. Andersson, H. Enroth

**Affiliations:** 1 Department of Infectious Diseases, Skaraborg Hospital, 541 85 Skövde, Sweden; 2 Department of Clinical Microbiology, Unilabs, Skaraborg Hospital, Skövde, Sweden; 3 CARe (Center for Antibiotic Resistance Research), Department of Infectious Diseases, Institute of Biomedicine, Sahlgrenska Academy at Gothenburg University, Gothenburg, Sweden; 4 Department of Clinical Molecular Microbiology, Unilabs, Skaraborg Hospital, Skövde, Sweden; 5 School of Biosciences, University of Skövde, Skövde, Sweden

**Keywords:** Influenza, Respiratory Syncytial Virus, Respiratory Virus, Viral Respiratory Infection, Acute Lower Respiratory Infection

## Abstract

The study aim was to investigate the prevalence and clinical relevance of viral findings by multiplex PCR from the nasopharynx of clinically septic patients during a winter season. During 11 weeks of the influenza epidemic period in January–March 2012, consecutive adult patients suspected to be septic (*n* = 432) were analyzed with cultures from blood and nasopharynx plus multiplex PCR for respiratory viruses on the nasopharyngeal specimen. The results were compared with those from microbiology analyses ordered as part of standard care. During the winter season, viral respiratory pathogens, mainly influenza A virus, human metapneumovirus, coronavirus, and respiratory syncytial virus were clinically underdiagnosed in 70% of patients positive by the multiplex PCR assay. During the first four weeks of the influenza epidemic, few tests for influenza were ordered by clinicians, indicating low awareness that the epidemic had started. Nasopharyngeal findings of *Streptococcus pneumoniae* and *Haemophilus influenzae* by culture correlated to pneumonia diagnosis, and in those patients laboratory signs of viral co-infections were common but rarely suspected by clinicians. The role of respiratory viral infections in patients presenting with a clinical picture of sepsis is underestimated. Specific antiviral treatment might be beneficial in some cases and may reduce spread in a hospital setting. Diagnosing viral infections may promote reduction of unnecessary antibiotic use. It can also be a tool for decisions concerning patient logistics, in order to minimize exposure of susceptible patients and personnel.

**Electronic supplementary material:**

The online version of this article (doi:10.1007/s10096-017-2990-z) contains supplementary material, which is available to authorized users.

## Introduction

### Respiratory tract infections

The respiratory tract is the most common focus of infection in septic patients. In adults with community-acquired pneumonia (CAP), a bacterial etiology can be established in about 25–50% of cases depending on definitions and methods used [1, 2]. *S. pneumoniae* is the most often found bacteria, followed by *H. influenzae* and *Mycoplasma pneumoniae*. More rarely found agents are *Legionella pneumophila*, *Chlamydophila pneumoniae,* and *Coxiella burnetti* [1, 3]. Several respiratory viruses may also cause severe respiratory disease, including CAP, mainly in children, but also in adults. This is well known for influenza A and B viruses, respiratory syncytial virus, coronavirus, human metapneumovirus, parainfluenza viruses types 1–3, adenovirus, enteroviruses, rhinovirus, and human bocavirus [3–5]. Indeed, viral infections are estimated to cause around 100 million annual cases of CAP worldwide [6]. Many of these viruses have seasonal variation patterns, causing epidemics, often with peaks during winter and early spring [7]. Viral respiratory infections may predispose for bacterial infections by damaging the respiratory epithelium as well as by viral-bacterial interactions. For example, influenza A virus can enhance the pathogenicity of *S. pneumoniae*, *Staphylococcus aureus* or *H. influenzae.* On the other hand it can inhibit the pathogenicity of others, such as *M. pneumoniae* and *C. pneumoniae* [8–11]. Using molecular techniques it has become evident that viral infection is present in around 25% of CAP, regardless of severity [1, 12, 13]. Viral co-infections in CAP has been shown to increase both disease severity and length of stay in hospital [14]. In patients with pneumonia requiring intensive care, mixed viral-bacterial infections have demonstrated the highest mortality rate in at least one study [13].

Commercial multiplex tests are continuously being developed, allowing for rapid etiological diagnosis of a wide range of respiratory viruses and bacteria [4, 15–18]. Diagnosing viral respiratory infections may help in reducing admissions, length of stay, use of antibiotic treatment and, in some cases, target antiviral treatment [14]. Testing is optimal during the first days of infection, when the viral load is high [19]. The clinical significance of a viral finding cannot always be determined. Some respiratory viruses, like rhinovirus, can persist in young children up to 6 weeks after a clinical infection [20], though persistence and long-term carriage seems to be less frequent in adults [1, 21].

### Sepsis

The definition and criteria for sepsis have changed over the years. Currently, sepsis is defined as a “life-threatening organ dysfunction caused by a dysregulated host response to infection” and said to be present if a patient has an infection and +2 points or more in the SOFA-score [22]. From a clinical viewpoint, a patient is suspected to be septic if there has been a sudden onset of chills and fever accompanied by abnormalities in vital signs, such as an increased respiratory rate, lowered oxygen saturation, tachycardia, hypotension, altered mentation, general malaise, and if laboratory findings such as an increase in leucocytes, C-reactive protein, lactate or procalcitonin support that suspicion. If so, broad-spectrum antibiotic treatment is started on clinical suspicion of bacterial sepsis and according to the preliminary diagnosis. Treatment is later modified according to the results of cultures, other microbiological detection methods, infection biomarkers, and imaging. In most cases, the origin and etiology of the infection cannot be established in the emergency department. Clinical symptoms and signs are helpful but not fully reliable. The inflammatory response in sepsis may itself cause symptoms and signs that can be misleading [23]. For example, a bacteremic urinary tract infection may present with predominating respiratory symptoms and signs [24]. This warrants broad testing early during care.

In this study conducted during the winter season, we pragmatically investigated the clinical relevance of nasopharyngeal viral and bacterial findings from clinically septic patients with suspected respiratory focus or sepsis of unknown origin.

## Materials and methods

### The severe sepsis study in Skaraborg

Over nine months, from September 2011 until June 2012, we performed a prospective, consecutive, epidemiologic study to investigate the incidence of community onset severe sepsis and septic shock in adults in Skaraborg, a rural area in Sweden with a population of 256,000. A single public hospital, Skaraborg Hospital, serves this population. Study inclusion criteria were: residents of Skaraborg 18 years or older who were treated within 48 h of admission with intravenous antibiotics on clinical suspicion of sepsis. The study was a “real life” study. No formal criteria for sepsis had to be fulfilled. Blood cultures were drawn from all patients before starting antibiotic treatment. Nasopharynx culture was performed on patients with a suspected focus in the respiratory tract or sepsis with unknown focus. Other cultures were done according to the clinical judgment of the treating physician. All patients were evaluated by protocol for the presence or development of severe sepsis or septic shock according to the Swedish definition and criteria [25]. The study was approved by the Regional Ethics Committee in Gothenburg (376–11).

The present study was part of the epidemiological study of severe sepsis, and was carried out during “the flu season” in patients admitted to the hospital from January 19 to March 26. Review of the complete patient records for all patients was performed according to protocol by one infectious diseases specialist (LRL). Clinical relevance was estimated from clinical notes on sudden onset of respiratory symptoms, dry or productive cough, shortness of breath, congestion, fever, imaging showing new infiltrates and when the discharge diagnosis contained a respiratory infection.

### Collection of patient samples

**Fig. 1:**
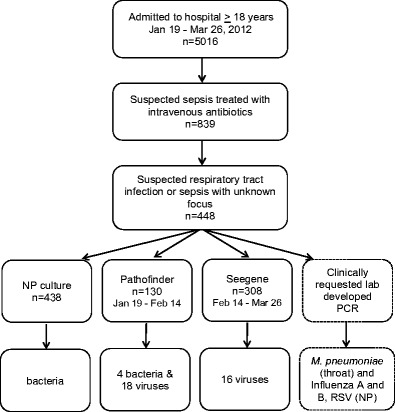
Out of 5,016 patients admitted to the hospital during the study period, 839 (16%) were treated with intravenous antibiotics on suspicion of sepsis. Around 50% of them were suspected to have a respiratory focus or an unknown focus of infection, sampled from the nasopharynx, and included in the study. Two different multiplex PCR tests were used for the study patients. In clinical use for all patients during the study period was a lab-developed PCR for influenza A and B virus and respiratory syncytial virus (triple test) as well as a lab-developed PCR for *M. pneumoniae* detection. *RSV* respiratory syncytial virus, *NP* nasopharynx

Sampling from the nasopharynx was performed using a flocked swab in universal transport medium (eSwab, Copan) on study patients suspected to be septic and to have a lower respiratory tract infection or sepsis with an unknown focus. Results obtained from this swab are comparable to those from nasopharyngeal aspirate [26]. Specimens were used for traditional NP-culture according to conventional methods and then stored at −20 °C. The samples were plated within 24 h after collection and plates were read after 24 and 48 h incubation. Target organisms were *S. pneumoniae*, *H. influenzae*, *M. catarrhalis*, beta-hemolytic streptococci groups A, C, and G. Growth of other species were reported only when observed in pure culture. All stored samples were analyzed within three months using either of two different viral respiratory multiplex PCR tests (Fig. 1).

Two sets of blood cultures (BacT/Alert, BioMerieux) were drawn from each patient before initiation of intravenous antibiotic treatment.

### Routine PCR analysis of respiratory infections

A separate flocked nasopharyngeal swab (eSwab, Copan) was used for routine testing of influenza A and B viruses, respiratory syncytial virus (triple viral test) and *M. pneumoniae* and collected at the discretion of the treating physician. These swabs were transported in 1 mL NaCl and used for in-house PCR tests. Results from the triple viral PCR tests were reported within a few hours but also used for comparison with the results obtained later from the samples collected and analyzed with the commercial tests (see below) as part of this study. Analysis of *M. pneumoniae* was performed on the nasopharyngeal samples by a probe-based lab-developed real-time PCR detecting the adhesin gene of *M. pneumoniae*. This PCR assay was run, analyzed, and interpreted on the Rotorgene instrument (Qiagen).

### Nucleic acid extraction

Nasopharyngeal samples from the original tubes were pre-pipetted to 96-plates in a Microlab Starlet (Hamilton Robotics, Switzerland) before extraction. Total nucleic acid extraction was performed with the MagNA Pure 96 instrument (Roche Applied Science) using the DNA and Viral NA small volume kit, the protocol Pathogen Universal, sample volume 300 μL and elution volume 100 μL [20]. Samples of extracted nucleic acids were mixed into the PCR reagents with a Qiagility (Qiagen, Hilden, Germany).

### Real-time multiplex PCR for respiratory viruses

An analysis of samples collected during the winter can be found in Fig. 1. From January 19 to February 14 (*n* = 130), a broad-spectrum commercial multiplex real-time PCR assay detecting 18 viruses and four bacteria was used (Respifinder SMART 22 kit, Pathofinder, Netherlands) [21]. Respifinder is designed to detect respiratory syncytial virus types A and B, adenovirus, human metapneumovirus, influenza A and B viruses, rhinovirus, enteroviruses, parainfluenza viruses types 1–4, human bocavirus, coronavirus types NL63, HKU1, 229E, and OC43, *M. pneumoniae*, *L. pneumophila, Bordetella pertussis,* and *C. pneumoniae.* This PCR assay was run, analyzed and interpreted on the Rotorgene instrument (Qiagen). One sample gave no results and was excluded from the study. In total, 129 multiplex results were used for the final analysis.

Samples collected from February 15 to March 26 (*n* = 308) were analyzed by a commercial multiplex real-time PCR assay detecting 16 respiratory viruses (Anyplex II RV 16, Seegene, Korea). This kit detects respiratory syncytial virus types A and B, adenovirus, human metapneumovirus, influenza A and B viruses, rhinovirus, enteroviruses, parainfluenza viruses types 1–4, human bocavirus, and coronavirus types NL63, 229E, and OC43 [22]. This PCR assay was run on the CFX96 Real-time PCR system (BioRad, France) and was analyzed and interpreted using “Seegene viewer” software (Seegene). Five samples were invalid and were excluded, leaving 303 results by the Anyplex II PCR for the final analysis. In total, 432 clinical NP samples were analyzed by multiplex PCR.

### Definition of pneumonia and respiratory tract infection

The Swedish Infectious Disease Society national guidelines define pneumonia as “symptoms or signs consistent with acute lower respiratory infection in combination with radiological findings compatible with this disease” (updated 2016, online and in Swedish only). A diagnosis of pneumonia in this study was made if there were symptoms or signs consistent with acute lower respiratory infection, and a finding within the first 48 h of a new infiltrate by chest X-ray or CT scan, and if no alternative diagnosis accounting for the new infiltrate was made during the stay in hospital. A diagnosis of respiratory tract infection was made if there were symptoms or signs consistent with acute respiratory infection but no X-ray findings compatible with pneumonia or if no imaging was performed and if there was no other explanation for the symptoms during the stay in hospital.

## Results

A total of 5,016 patients were admitted to the hospital during the study period. The average age was 71 years and 50.1% were men. Out of those admitted, 839 (16.7%) received intravenous antibiotic treatment within 48 h and were included in the larger epidemiological sepsis study. The average age was 69 years and 54.5% were men. From 438 out of 839 (52.2%) patients a nasopharyngeal swab was collected and 432 could be evaluated by both culture and multiplex PCR for bacteria or respiratory viruses.

### Nasopharyngeal findings by real-time multiplex PCR

Multiplex PCR testing detected 166 viruses in 158 of 432 patients (37%) (Table 1) of whom 60% were men, with an average age of 70 years, and 40% were women, average age 73 years. No influenza B virus was found in study samples. Two samples positive for influenza A virus by routine testing could not be confirmed by multiplex PCR.

**Table 1:** Findings in nasopharyngeal swabs by multiplex PCR from 432 patients with suspected sepsis

Pathogen	Number of findings	Percent of total (%)
Influenza A virus	96	22
Human metapneumovirus	23	5
Coronavirus types OC43, 229E, and HKU1	19 (14, 2, 3)	4
Respiratory syncytial virus types A and B	12 (6, 6)	3
Rhinovirus and enteroviruses	10	2
Parainfluenza viruses types 1, 2, 3, and 4	3 (2, 1)	0.6
Human bocavirus	2	0.4
Adenovirus	1	0.2
*Mycoplasma pneumoniae*	5	1
Total	171	

In a study of 432 patients with suspected sepsis during the winter season in 2012, 166 viruses were found in 158 patients by multiplex PCR on specimens from the nasopharynx. In eight patients there were double findings, six including influenza A virus, three including human metapneumovirus and one including respiratory syncytial virus. Out of the first 129 patients tested by the Pathofinder multiplex PCR, five were positive for *M. pneumoniae*

**Fig. 2:**
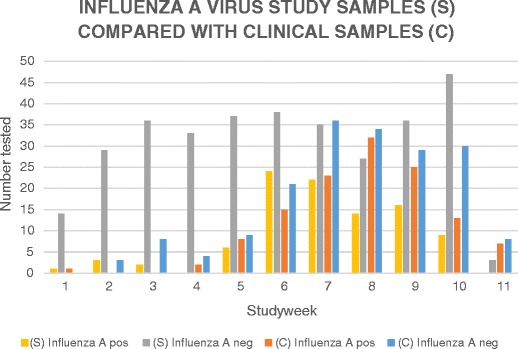
Results for influenza A virus testing between January 19 and March 26, 2012. Samples from study patients with suspected sepsis are compared with all clinically requested samples for influenza A virus in all hospitalized patients. There were very few clinical requests (C) for influenza A virus testing during the first four weeks of the de facto influenza season, indicating a lack of systemic awareness and clinical suspicion even during a rapid escalation in cases

Influenza A virus was the most common finding, detected in 96/158 (61%) of study patients. During the same period, clinicians ordered the triple viral test for influenza A and B viruses and respiratory syncytial virus in 308 patients. Of those, 126 (41%) were positive for influenza A virus, 1 (0.3%) for influenza B virus and 7 (2%) for respiratory syncytial virus. During the first three study weeks, only one clinically requested test was positive for influenza A virus compared with six in the study group. During the first four weeks of the influenza epidemic there were few clinically requested tests for influenza virus. This indicates that early in the influenza epidemic, awareness among clinicians that the flu season had started was low (Fig. 2). The routine triple viral test for influenza A and B viruses and respiratory syncytial virus available to clinicians was requested in only 56 of 107 (54%) patients who turned out positive for those viruses in the study analysis.

The Pathofinder multiplex PCR used for the first 129 patients of the study was positive for *M. pneumoniae* in five cases. Two of these patients also tested positive in the clinically available test. During the latter part of the study, when PCR for *M. pneumoniae* was not included in the multiplex PCR, there was one additional patient positive for *M. pneumoniae* by routine PCR testing.

### Nasopharyngeal findings by culture

By conventional culture from the nasopharynx culture there were 101 out of 432 (23.4%) patients positive for any bacteria (Table 2).

**Table 2:** Bacterial findings in nasopharyngeal swabs by culture and multiplex PCR from 432 patients with suspected sepsis

Bacteria	Number of findings	Percent (%) of findings
*S. pneumoniae*	19	17
*H. influenzae*	18	16
*M. catarrhalis*	31	28
*S. aureus*	14	11
*S. pyogenes*(3) and *dysgalactiae*(2)	5	5
Enterobacteriacae	5	5
*N. meningitidis*	2	2
*Corynebacterium liquefaciens/propinquum*	8	7
Other	2	2
*M. pneumoniae* (by multiplex PCR)	5	5
Total	109	

In a study of 432 patients with suspected sepsis during the winter season in 2012, 109 bacteria were found in the nasopharynx in 104 patients. Culture diagnosed 104 of those. The Pathofinder multiplex PCR was used for the first 129 of 432 patients revealed five cases of *M. pneumoniae.* In five cultures there were double bacterial findings. Three had *H. influenzae* and *M. catarrhalis*. Two positive for *M. pneumoniae* by multiplex-PCR also had *H. influenzae* and *N. meningitidis*, respectively

### Mixed bacterial and virological findings

In total, 50 of 432 (12%) patients were positive for both a respiratory virus and bacteria. The bacteria most often associated with a viral respiratory pathogen were *S. pneumoniae* in 14 of 50 (28%), *M. catarrhalis* in 14 (28%), *S. aureus* in 7 (14%) and *H. influenzae* in 6 (12%). The most frequently found viruses were influenza A virus in 27 (54%), human metapneumovirus in 10 (20%), and respiratory syncytial virus in 4 (8%). *S. pneumoniae* was found together with a virus in 14 of 19 samples, *H. influenzae* in 6 of 17, *M. catarrhalis* in 14 of 28 and *Staphylococcus aureus* in 7 of 14 (Table 3).

**Table 3:**
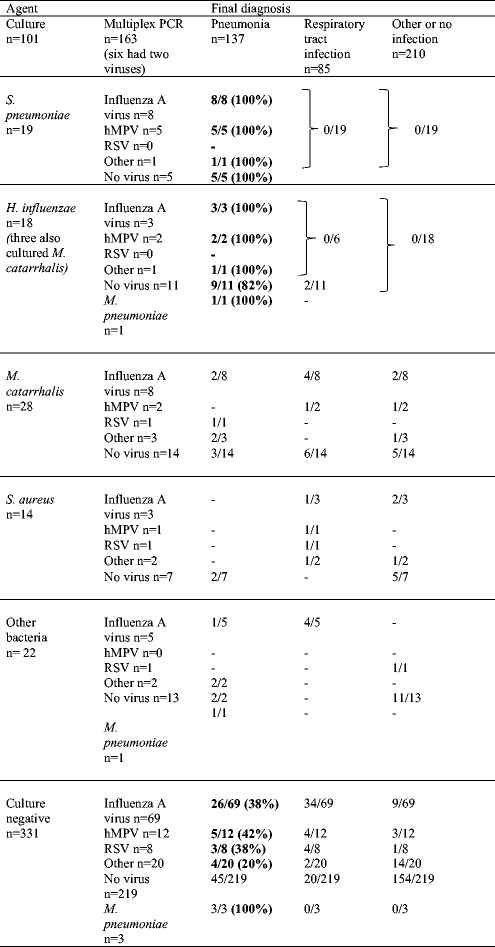
Correlation between results by culture and multiplex PCR in the nasopharynx to final diagnosis in adult patients (*n*) suspected to have sepsis

During the winter season in 2012, 432 patients with suspected sepsis were tested from the nasopharynx by culture and multiplex PCR for respiratory viruses. Finding of *S. pneumoniae* or *H. influenzae* strongly correlated to pneumonia. In 75% of patients with *S. pneumoniae* there was also a viral finding, mainly influenza A virus and human metapneumovirus. Apart from the viral findings, the Pathofinder Multiplex PCR test detected five cases of *M. pneumoniae.* All five had pneumonia. One also grew *H. influenzae* and had a rapidly fatal course. One also grew *Neisseria meningitidis* and had a mild course

*hMPV* = human metapneumovirus; *RSV* = respiratory syncytial virus types A and B

### Blood cultures

Two sets of blood cultures were drawn before initiation of intravenous antibiotic treatment in all 432 patients. Of those, 35 patients (8%) were positive for a true bacterial pathogen [27]. The bacterial species were *S. aureus *(*n* = 12), *E. coli *(n = 8), *S. pneumoniae* (*n* = 5), *Klebsiella pneumoniae *(*n* = 4), *S. pyogenes* (*n* = 2), and others species in four patients. In the overall nine-month epidemiological study, 313 of 2,472 (12.6%) blood cultures were positive for a true pathogen.

### Clinical diagnoses in patients with suspected sepsis and microbial findings in the nasopharynx

Of the 158 patients with a finding of one or two respiratory viruses, the respiratory tract was the initially suspected focus of infection in 70%. In 18% it was sepsis with unknown focus, and in 12% it was some other focus (Table 4).

**Table 4:** Initially suspected focus of infection in 158 septic patients with viral findings in the nasopharynx by multiplex PCR

Virus (N = 158)	Initially suspected focus (*n*)			Suspected focus other than the respiratory tract, *n *(%)	Initial clinical suspicion of “influenza” or “virosis”, *n *(%)
	Respiratory tract, *n *(%)	Sepsis UNS, *n* (%)	Other, *n* (%)		
Influenza A virus, *n* = 96	79	13	4	17 (18)	35/96 (36)
Human metapneumovirus, *n* = 22	14	7	1	8 (36)	5/22 (23)
Respiratory syncytial virus, *n* = 11	8	1	2	3 (27)	3/11 (27)
Coronavirus, *n* = 16	6	3	7	10 (63)	1/16 *(6)*
Rhinovirus or enteroviruses, *n* = 9	3	4	2	6 (67)	0/9 *(0)*
Other, *n* = 4	1	-	3	3 (75)	1/4 *(25)*
Total, *n* = 158	111/158 (70)	28/158 *(18)*	19/158 *(12)*	47/158 (30)	45/158 (30)

Despite being in the winter season, a viral etiology or co-etiology was initially considered in only 30% of the patients with suspected sepsis. In 30% the initial focus of the infection was believed to be some other than the respiratory tract, mainly “Sepsis with unknown focus”. Eight patients had double viral findings. Six were found together with influenza A virus and were counted as influenza A virus. Two were found together with human metapneumovirus and were counted as human metapneumovirus

According to the patient records, there was an initial clinical suspicion of a respiratory viral infection as part of the clinical picture in only 30% of the cases, even though the study occurred well into the known influenza season. In the emergency department, only 36% of all study patients positive for influenza A virus were initially suspected to have “influenza” or “virosis”. During the time in hospital, another 18%, altogether 54%, were clinically suspected to have influenza and were tested by routine analysis. Out of 33 patients positive for either human metapneumovirus or respiratory syncytial virus, only 35% were clinically tested on suspicion of a viral infection. Yet, in the patient records, respiratory symptoms were documented in as many as 87 of 96 (90%) patients positive for influenza A virus and in all 33 positive for human metapneumovirus or respiratory syncytial virus. Only in 9 of 96 patients with fever testing positive for influenza A virus, there was no documentation of respiratory tract symptoms. Findings of rhinovirus, enteroviruses, coronavirus or human bocavirus were less often linked to respiratory disease, and more often found in patients with infections outside the respiratory tract, or with no infection at all (Table 3).

Out of 158 patients positive for any viral agent, 17 fulfilled at least one of the Swedish criteria for severe sepsis or septic shock. Fourteen of those were in the group with a new pulmonary infiltrate.

Bacterial findings of *M. pneumoniae* (*n* = 5),*S. pneumoniae* (*n* = 19) and *H. influenzae* (*n* = 18) correlated to pneumonia in 39 of 41 (95%) cases. One patient positive for *H. influenzae* had a co-infection with *M. pneumoniae*, suffered from severe sepsis, and died after one day in the ICU. Another positive for *M. pneumoniae* was co-infected with *Neisseria meningitidis* and had a clinically mild course. Findings of *M. catarrhalis, S. aureus* (no MRSA), Enterobacteriaceae spp., beta-hemolytic streptococci groups A, C, or G, or other bacteria, were less often associated with pneumonia.

Pneumonia, as indicated by a new infiltrate on chest X-ray or CT scan, was demonstrated in 137 of 432 study patients, with a median age of 73 years, whereby 55% were men. In another eight patients it could not be determined whether there was a new infiltrate, and in 28 patients no chest X-ray was performed. In patients with pneumonia, 91 of 137 were positive in the nasopharynx for some respiratory virus or bacteria. The most common agents were influenza A virus (*n* = 40), *S. pneumoniae* (*n* = 19), *H. influenzae* (*n* = 16), human metapneumovirus (*n* = 12), *M. pneumoniae* (*n* = 5), and respiratory syncytial virus types A or B (*n* = 4). In 22 patients there was a co-finding of any of those bacteria plus any of those viruses and in one patient there was a co-finding of *H. influenzae* and *M. pneumoniae* (Table 3).

## Discussion

We found that viral infections were often neglected during this population-based “real life” study of suspected septic patients. The study was performed during the winter period, referred to as “the flu season”, when respiratory viral infections are most prevalent. This should have resulted in increased clinical suspicion. Yet, during the first four weeks of the influenza epidemic, very few clinical samples are requested. In this material, a viral respiratory infection was initially suspected by clinicians in only 30% of patients with viral findings by multiplex PCR. This was especially true when CRP was over 100 mg/L, or if there was a new infiltrate on the chest X-ray indicating pneumonia. This underestimation may lead to nosocomial spread or outbreaks of viral respiratory infections, as we have previously experienced in our own hospital. It may also lead to overuse of antibiotics, as well as underuse of antivirals, especially in risk groups that might benefit from such treatment.

As in a comprehensive study on bacterial–viral respiratory tract illness over three winter seasons by Falsey et al. in 2013 [28], influenza A virus was the most common viral finding, appearing in study samples almost two weeks earlier than in clinical samples. In only 35 of 96 cases of influenza A virus infection (36%) was influenza virus initially suspected as sole cause or contributing factor to the acute illness.

Respiratory syncytial virus and human metapneumovirus may cause critical respiratory illness and pneumonia, not only in children, but also in elderly. For example, human metapneumovirus was found to be the causative agent in an outbreak of pneumonia among elderly at an institution in the Netherlands [29]. In this study, human metapneumovirus was a slightly more common finding than respiratory syncytial virus, especially in patients with long history of fever and respiratory tract congestion, combined with radiological signs of pneumonia.

Nasopharyngeal culture is generally discouraged or not recommended for etiological diagnosis of pneumonia. However, in a Swedish study by Strålin et al. in 2006 [30], there was a good correlation between nasopharyngeal findings of these bacteria and the etiology of pneumonia, as has been seen in previous Swedish studies. In our study of patients in the emergency department suspected to be septic, there was a strong correlation between nasopharyngeal findings of *S. pneumoniae* or *H. influenzae* and X-ray findings of a new infiltrate, indicative of pneumonia. More so, these bacteria were not found in the nasopharynx of any of the 210 patients with a non-respiratory infection or no infection, and they were rarely found in patients with a respiratory tract infection but not pneumonia. The study results imply that nasopharyngeal findings of *S. pneumoniae* and *H. influenzae* in sepsis patients should be considered carefully for patient treatment. A recently published paper by Bjarnason et al. in 2017 [31] demonstrates a good correlation between real-time PCR findings of *S. pneumoniae* or *H. influenzae* to pneumonia diagnosis in adults, which also builds support for a clinical relevance of these upper respiratory bacterial findings.

Co-infections of bacteria and respiratory viruses, mainly *S. pneumoniae* and influenza A or respiratory syncytial virus, are found in 3–40% of patients with CAP, depending on diagnostic methods used, with the higher end reflecting studies in which nasopharyngeal culture is included for etiological diagnosis [1, 2]. Using nasopharyngeal sampling only, we found indications of viral-bacterial co-infections in 28 of 137 (20%), a proportion we believe to be an underestimation. As in the study by Falsey et al. in 2013 [28], *S. pneumoniae* was the bacteria most often associated with pneumonia and a viral co-infection. As many as 75% of patients with pneumonia and *S. pneumoniae* in the nasopharynx were positive for a respiratory virus, mainly influenza A virus, but also human metapneumovirus. The two youngest patients, with pneumonia and severe sepsis, aged 37 and 42 years respectively, were both positive for *S. pneumoniae* and human metapneumovirus in the nasopharynx. No other pathogens could be demonstrated by routine cultures.

In the clinical setting it is often difficult to determine whether a patient with respiratory symptoms has a viral infection, a bacterial infection, or a mixed viral-bacterial infection. No constellation of clinical symptoms, vital signs, biomarkers (such as white blood cell count, C-reactive protein, or procalcitonin) have adequate sensitivity and specificity. New tools to improve predictions of patient benefit from antibiotic treatment are urgently needed. Recently, whole blood analysis for the identification of host gene activation profiles has been able to discriminate viral infections from bacterial infections with high accuracy in severely ill infants, as described by Herberg et al. in 2016 [32]. In adults with lower respiratory tract infections, a similar technique seems able to discriminate viral from bacterial infections much better than procalcitonin, as shown by Suarez et al. in 2015 [33]. In the same study mixed viral-bacterial infections also elicited a characteristic gene activation profile.

Our study supports increased testing for respiratory viruses in patients believed to be septic, especially those presenting with respiratory tract symptoms. With current technology, results can be obtained within a few hours and have an impact on clinical decisions and patient logistics in the emergency department. Cost effectiveness should be investigated. A viral diagnosis may not only lead to fewer admissions and less antibiotic treatment if bacterial pneumonia is suspected or demonstrated, but may also decrease viral exposures for admitted patients. Patients with influenza A or B may benefit from antiviral treatment alone or in conjunction with antibacterial treatment, if bacterial pneumonia is suspected or demonstrated, perhaps even reducing viral contagiousness. Even in neutropenic patients, a viral finding and a favorable outcome in the first few days may safely allow for discontinuation of antibiotic treatment [34].

This study has several limitations. It is a single-center study performed during one winter period only. The study was primarily not an etiological study of respiratory tract infections. We did not take ongoing antibiotic treatment into account. Only routine sampling from the nasopharynx was performed, albeit using a flocked swab for better yield. If sputum or nasopharyngeal aspirates had been analyzed with molecular techniques, we would have expected a higher yield for both bacteria and viruses [2, 30]. A further weakness is the open inclusion based on clinical suspicion of sepsis only, without specific criteria. Yet another was the subjectivity involved in deciding the relevance of the findings. What role do the viral findings play, both alone or in conjunction with bacterial findings? We can only show correlations between findings and clinical entities, yet the majority of findings do seem to correlate well to respiratory tract infections. Therefore, we believe that our conclusion, that significant viral disease in severely ill patients is underdiagnosed by clinicians, is warranted. Diagnosing these infections early may be of help for the clinical decision making process and thereby for the patients.

## Conclusion

We found that viral respiratory pathogens were underdiagnosed in severely ill patients suspected to be septic, many presenting with clinical symptoms from the respiratory tract. This may lead to nosocomial spread of viral respiratory infections, unnecessary use of antibiotics and underuse of antivirals in the hospital setting.

Furthermore, we found a correlation between nasopharyngeal findings of *S. pneumoniae* or *H. influenzae* and pneumonia in patients with suspected sepsis. Though sensitivity is low, specificity is high. In light of disease severity and low analysis cost, this study can support the use of admission nasopharyngeal culture as part of diagnostic bundles for sepsis patients. An extended use of molecular tests could improve diagnosis, patient care and clinical outcome in patients with suspicion of sepsis, while decreasing risk for nosocomial infections.

## Electronic supplementary material

ESM 1 (XLSX 39 kb)
